# ETHICAL CONSIDERATIONS UNDERLYING ACP FOR ACCOMPANIED OLDER ADULTS WITH COGNITIVE IMPAIRMENT

**DOI:** 10.1093/geroni/igad104.0043

**Published:** 2023-12-21

**Authors:** Martha Abshire Saylor, Cynthia Boyd, Sydney Dy, Valecia Hanna

**Affiliations:** Johns Hopkins University School of Nursing, Baltimore, Maryland, United States; Johns Hopkins School of Medicine, Baltimore, Maryland, United States; Johns Hopkins School of Public Health, Baltimore, Maryland, United States; Johns Hopkins School of Public Health, Baltimore, Maryland, United States

## Abstract

While clinicians understand the importance of ACP, it is difficult to overcome logistical and ethical challenges in the limited time providers have in primary clinic appointments with older adults with ADRD and other complex co-morbidities. Ethical/moral dilemmas faced by patients, families, and clinicians are reflected in the four principles of biomedical ethics including autonomy, beneficence, nonmaleficence, and justice. While ethical challenges are recognized there is a lack of real-world evidence to guide clinicians or family caregivers in ACP for older adults with ADRD based on the reality of complex patient and family dynamics. Throughout the SHARE trial, facilitated ACP conversations with trained ACP facilitators, older adult patients with cognitive impairment, and their family caregiver were audio recorded and transcribed. The purpose of this presentation is to expand our understanding of best practices for facilitating ACP conversations among persons with ADRD and their family caregivers, guided by medical ethics and the Learning Health Care System Ethics Framework. We draw on the unique dataset of recorded ACP conversations involving older adults with severe cognitive impairment (N=92, average age=88 years), family caregivers, and trained ACP facilitators. We qualitatively coded transcripts for ethical considerations of ACP conversations and conducted the thematic analysis. We will present major themes related to ethical considerations in facilitating ACP conversations among persons with moderate to severe ADRD and their family caregivers. We will provide a discussion framed in balancing patient, family, and provider needs within a Learning Health Care System.

